# Preferential Control of Basal Dendritic Protrusions by EphB2

**DOI:** 10.1371/journal.pone.0017417

**Published:** 2011-02-25

**Authors:** Matthew S. Kayser, Anderson C. Lee, Martin Hruska, Matthew B. Dalva

**Affiliations:** Department of Neuroscience, University of Pennsylvania School of Medicine, Philadelphia, Pennsylvania, United States of America; Centre national de la recherche scientifique, University of Bordeaux, France

## Abstract

The flow of information between neurons in many neural circuits is controlled by a highly specialized site of cell-cell contact known as a synapse. A number of molecules have been identified that are involved in central nervous system synapse development, but knowledge is limited regarding whether these cues direct organization of specific synapse types or on particular regions of individual neurons. Glutamate is the primary excitatory neurotransmitter in the brain, and the majority of glutamatergic synapses occur on mushroom-shaped protrusions called dendritic spines. Changes in the morphology of these structures are associated with long-lasting modulation of synaptic strength thought to underlie learning and memory, and can be abnormal in neuropsychiatric disease. Here, we use rat cortical slice cultures to examine how a previously-described synaptogenic molecule, the EphB2 receptor tyrosine kinase, regulates dendritic protrusion morphology in specific regions of the dendritic arbor in cortical pyramidal neurons. We find that alterations in EphB2 signaling can bidirectionally control protrusion length, and knockdown of EphB2 expression levels reduces the number of dendritic spines and filopodia. Expression of wild-type or dominant negative EphB2 reveals that EphB2 preferentially regulates dendritic protrusion structure in basal dendrites. Our findings suggest that EphB2 may act to specify synapse formation in a particular subcellular region of cortical pyramidal neurons.

## Introduction

Mature cortical neurons are decorated with thousands of dendritic spines. These structures are the site of the majority of excitatory synapses and are thought to be critical for the generation and expression of synaptic plasticity [1], [2]. Spine morphology is dynamic during development and abnormal in a number of cognitive and neurodegenerative disorders such as autism and Alzheimer's disease [1]. Multiple trans-synaptic signals have been identified that control aspects of neuronal synapse and dendritic spine formation [2], and are implicated in neuropsychiatric abnormalities [3], [4]. A central question is whether these various proteins direct the formation of synapse subtypes, for instance by regulating development of contacts in a specific population of inputs.

Cortical pyramidal neurons have stereotyped morphology, with long apical dendrites that project to the cortical surface and short branched basal dendrites that remain largely within the same cortical layer as the cell soma [5], [6]. Synaptic inputs onto cortical neurons appear to be spatially segregated in a subcellular manner, with projections from particular cortical layers or areas of brain specifically synapsing on either the apical or basal portion of the dendritic arbor [7], [8]. These regions of the dendritic tree also have distinct functional significance with regard to integration of inputs, excitability, and plasticity [6]. In particular, basal dendrites receive a large proportion of the excitatory glutamatergic inputs impinging on cortical pyramidal cells, and dendritic spikes in basal dendrites rely more on NMDARs in comparison to voltage-gated channels in much of the apical tree [6], [9], [10]. Although cortical pyramidal neurons have specialized morphology and channel distribution, it is not known whether different mechanisms guide the formation of synaptic structures in specific parts of the dendritic arbor.

EphBs are one class of transmembrane signaling molecules involved in synapse and spine formation. Eph receptors are the largest known family of receptor tyrosine kinases in the mammalian genome, and are divided into A and B subclasses based on affinity for their membrane-associated ligands, ephrin-As and ephrin-Bs [11], [12]. EphB2 is found at synapses in cortex [13] and EphB signaling mediates multiple aspects of neuronal synapse development including dendritic filopodia motility [14], spine formation [14], [15], [16], [17], NMDA receptor clustering and function [18], [19], [20], [21], and presynaptic maturation [22], [23]. EphB-dependent regulation of dendritic filopodia motility and spine formation relies on forward signaling through guanine nucleotide exchange factors (GEFs) and other downstream molecules such as p21 activated kinase (PAK) that modulate the actin cytoskeleton [14], [17], [24], [25], [26]. *In vitro*, activation of EphB2 results in more spines and fewer filopodia, while overexpression of a dominant negative EphB2 construct that blocks EphB forward signaling results in fewer spines and more filopodia [15], [17], [25]. Interestingly, the role of EphB in filopodia motility and spinogenesis is specific to certain developmental windows, triggering increased motility early and spine formation/stability later in development [14]. While this temporal specificity is well-delineated, it remains unclear whether a spatial or anatomical specificity exists as well.

Previous work in cultured neurons and hippocampus or cortex of EphB1/EphB2/EphB3 triple knockout mice has demonstrated EphBs are required for the formation of many dendritic spine synapses [16], [22], but has not explored whether changes occur in a particular subcellular distribution. Here we examine how modulation of EphB2 in single cortical neurons in brain slice effects dendritic protrusion formation, and investigate whether these changes specifically impact apical or basal dendrites. During the same developmental period when loss of EphBs results in decreased synapse density, we find that EphB2 knockdown causes loss of dendritic spines and filopodia. Wild-type EphB2 overexpression increases spine density while disruption of EphB2 forward signaling results in increased filopodia density. These effects are observed preferentially in the basal dendritic arbor of cortical neurons, suggesting a role for EphB2 in dendritic protrusion structure in a specific subcellular domain.

## Results

**Figure 1:**
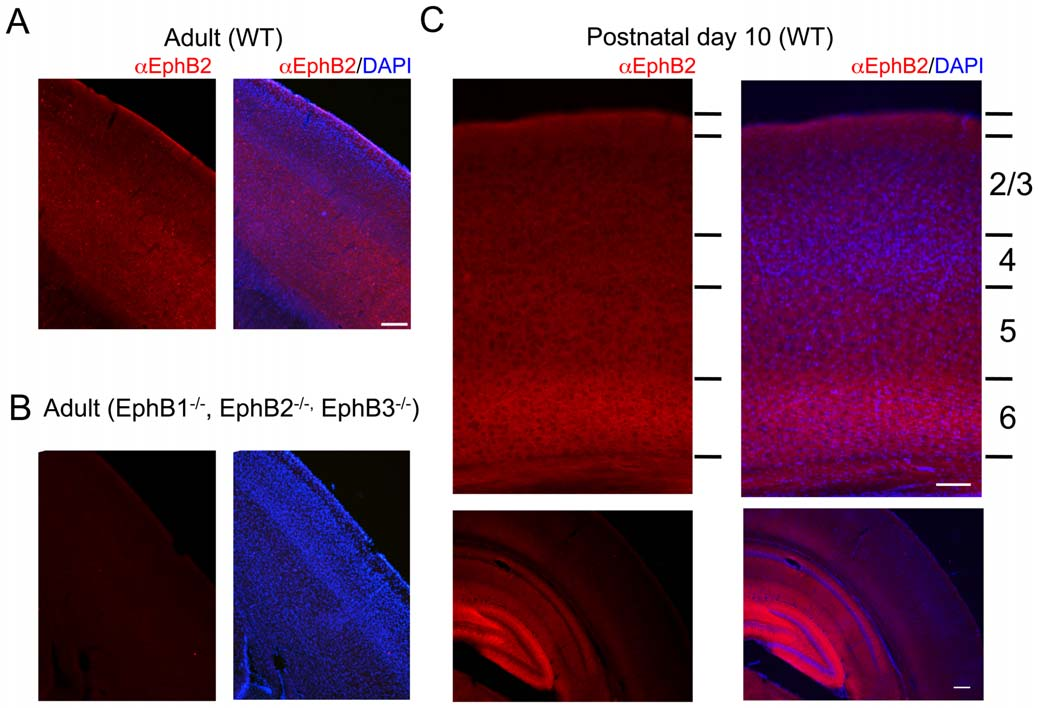
EphB2 is expressed in the developing and adult mouse cortex. **A**. Anti-EphB2 immunostaining of a sagittal brain section from adult mouse cortex. B. Anti-EphB2 immunostaining of a sagittal brain section from adult mouse cortex in an animal lacking EphB1-3. C. Immunostaining of a coronal brain section from P10 mouse cortex. Panels on left show EphB2 immunoreactivity in red. Right panels are merged images of EphB2 immunoreactivity and DAPI labeled nuclei in blue. In C, bottom panels show a lower magnification view of EphB2 immunostaining in the cortex and hippocampus of P10 animals. Scale bars = 100 µm in panels A, B, and upper C. Lower C scale bar = 200 µm.

**Figure 2:**
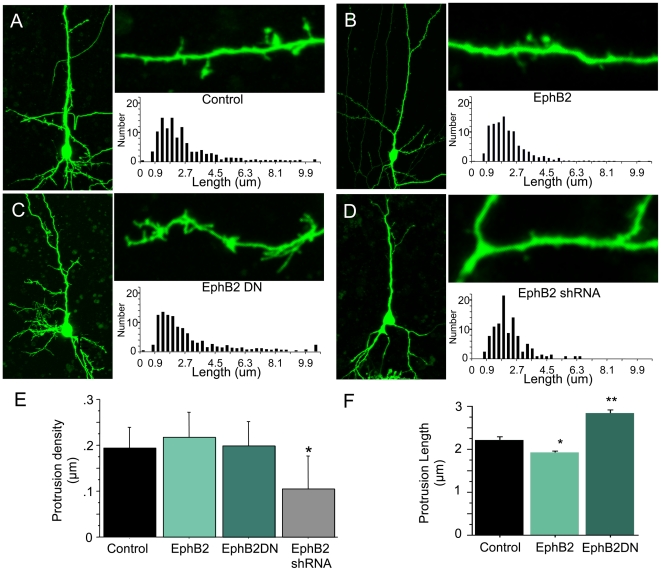
Alterations in EphB2 signaling and expression control dendritic protrusion length in brain slice culture. A. Example of 4DIV layer 2/3 control cortical neuron in brain slice culture transfected with EGFP. Images collected with a two-photon microscope Leica TSP-2 and z-stack generated using NIH ImageJ. Adjacent, a high magnification view of a segment of dendrite containing both spines and filopodia. Graph shows distribution of lengths of protrusions in control neurons. B. As in A, but cortical neuron transfected with EGFP and wild type EphB2 expression constructs. Quantification indicates an increased proportion of shorter protrusions. C. As in A, but cortical neuron transfected with EGFP and EphB2DN expression constructs. Quantification indicates an increased proportion of longer protrusions. D. As in A, but cortical neuron transfected with shRNA targeting EphB2. Quantification indicates an overall decrease in protrusion number. E. Quantification of the overall density of protrusions in analyzed neurons (Control n = 19, EphB2 n = 17, EphB2DN n = 15, EphB2shRNA n = 7 neurons). F. Quantification of the overall length of dendritic protrusions in Control (n = 654 protrusions), EphB2 (n = 1141), and EphB2DN (n = 750). Error bars indicate standard error. *p<0.005, ** p<0.001.

To examine how alterations in EphB2 signaling in individual neurons impacts dendritic protrusion formation in cortex, we transfected neurons in organotypic brain slice cultures of rat cortex with constructs expressing wild-type or dominant negative EphB2 along with green fluorescent protein (EGFP). Organotypic slice culture allows manipulation of EphB signaling while leaving neurons in an environment that maintains cortical layer structure, neuronal polarity, and many functional neuronal circuits. The dominant negative EphB2 (EphB2DN) lacks the intracellular domain of the protein, and has been shown previously to block EphB forward signaling without impacting tyrosine phosphorylation of other receptors like TrkB [18]. Cortical neurons in layer 2/3 or 5 of cultured brain slices transfected with GFP at 1-2DIV were imaged with 2-photon microscopy at 4-7DIV and dendrite protrusion length measured using custom written ImageJ (NIH) plugins. We focused on this period of development because immunostaining of cortical brain sections revealed that EphB2 is expressed in the neuropil of cortex at this time (Figure 1), with the highest level of expression found in the subgranular layers. Consistent with previous reports [19], [20], [27], we find that EphB2 expression is maintained in mature brain in cortex and hippocampus (Figure 1). In dissociated neuronal cultures, manipulation of EphB2 expression and signaling induces a shift in dendritic protrusion structure between spines and filopodia [15], [17], and in some preparations can result in alterations of overall dendritic protrusion density [25]. Therefore, we first asked whether expression of EphB2 and EphB2DN might also alter the number of dendritic protrusions in brain slices. Interestingly, neither overexpression of EphB2 nor EphB2DN resulted in a significant change in the overall density of dendritic protrusions (control, 0.194±0.022 protrusions/µm; EphB2, 0.217±0.026 protrusions/µm; EphB2DN, 0.198±0.025 protrusions/µm), suggesting that in complex tissue the role of EphB may differ in some aspects from its functions in dissociated neurons (Figure 2E).

While genetic deletion of EphBs from mouse brain has been found to result in fewer dendritic spines [16] and excitatory synapses [22], less is known regarding how reduced EphB expression in individual neurons in a more intact preparation impacts dendritic protrusion length. To study this question we used a previously characterized shRNA construct [14] that reduces the expression of EphB2 in neurons, can be rescued by expression of shRNA insensitive EphB2 constructs, and has no effect on neurons from EphB triple knockout mice (EphB1-/-, EphB2-/-, EphB3-/-). Consistent with previous work in dissociated neurons and in mice lacking EphB1-3 [16], [22], shRNA knockdown of EphB2 expression did result in a ∼50% reduction in dendritic protrusion density (control, 0.194±0.022 protrusions/µm; EphB2 shRNA, 0.104±0.030 protrusions/µm; p<0.05) (Figure 2). Together with results from EphB2 and EphB2DN overexpression, these findings indicate that while dendritic protrusions are not maintained in normal numbers in the absence of EphB expression, increasing EphB expression or interfering with downstream signaling does not simply result in addition of new protrusions of a particular type.

Although overall protrusion density was not changed, we next asked whether expression of either EphB2 or EphB2DN in individual cortical neurons in slice culture might result in a shift in dendritic protrusion length. Changes in the length of dendritic protrusion would suggest that EphBs might alter the maturation or structure of dendritic protrusions rather than their overall number. We found that expression of EphB2 resulted in a significant decrease in the average length of dendritic protrusions, while EphB2DN resulted in a significant increase in the length of protrusions (control, 2.22±0.06 µm; EphB2, 1.91±0.04 µm; EphB2DN, 2.49±0.08 µm; p<0.005, Figure 2F). These data indicate that EphB2 signaling can regulate the length of dendritic protrusions in organotypic slice culture, and are consistent with previous findings that EphB2 regulates the maturation of dendritic protrusions [14], [15], [17].

**Figure 3:**
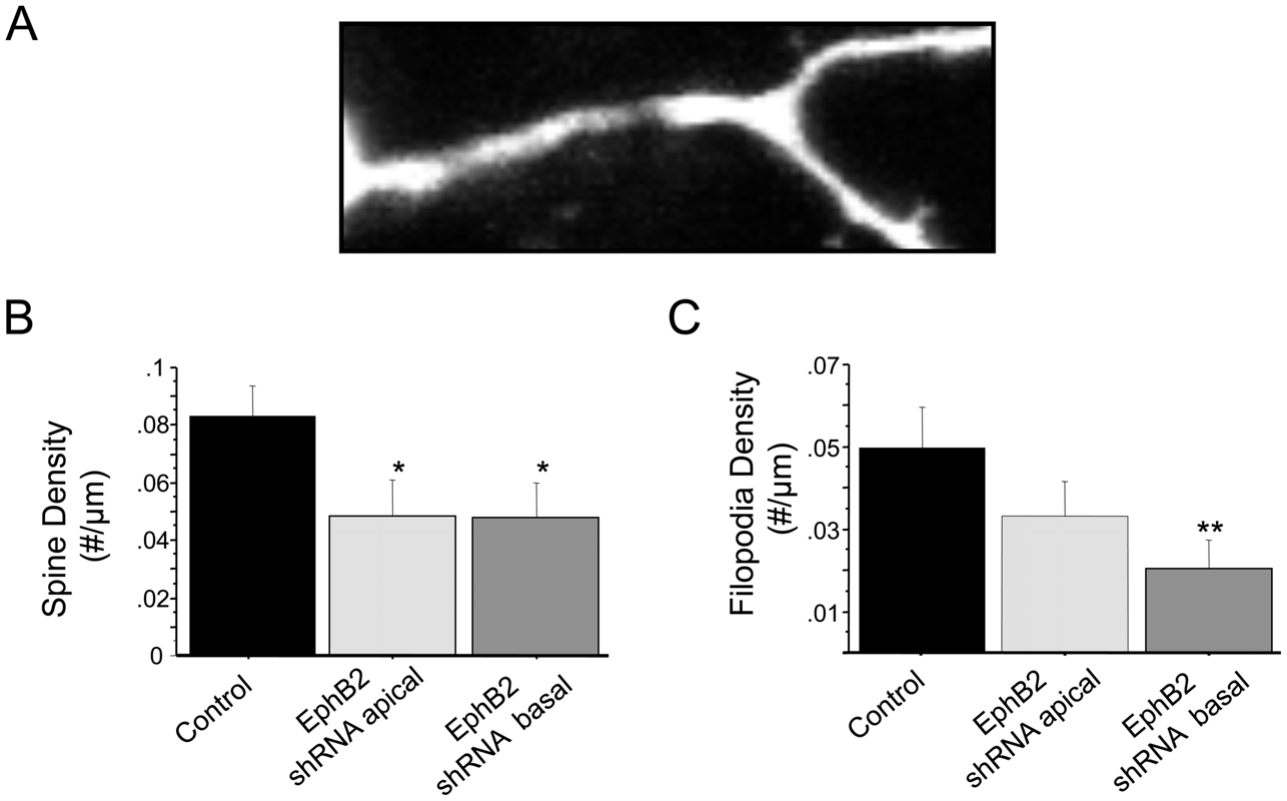
EphB2 knockdown in neurons in cortical brain slice causes uniform loss of spines and specific loss of filopodia in basal dendrites. A. High magnification view of two-photon image of a segment of a dendrite in cortical neuron transfected with EGFP and EphB2 shRNA. B. Quantification of spine density in control neurons (n = 16) and neurons transfected at 1–2 DIV with EphB2 shRNA and imaged at 4-7DIV, divided by region of dendritic tree (apical: n = 9; basal: n = 9). C. Quantification of filopodia density in control neurons (n = 16) and neurons transfected with EphB2 shRNA, divided by region of dendritic tree (apical: n = 9; basal: n = 9). Error bars indicate standard error. Scale bar = 2 µm; *p<0.05.

To investigate whether EphBs might play a role in determining the structure of dendritic protrusions in either apical or basal dendritic arbors specifically, we first examined how knockdown of EphB2 impacts the density of dendritic spines and filopodia in each region of the dendritic tree. Neurons in cortical brain slices were transfected at 2DIV with GFP and a previously characterized shRNA that selectively targets EphB2 [14]. Neurons were then imaged at 4-7DIV, and the density of various morphological classes of dendritic protrusions determined. Importantly, expression of the shRNA used to knockdown EphB2 has no effects in neurons from mice lacking EphBs, indicating that its effects are specific [14]. Using custom ImageJ plugins, we scored protrusions in the apical and basal dendrites of cortical neurons as spines or filopodia. Each protrusion was marked and its location within the image recorded. To avoid ambiguities in our classifications, we labeled a protrusion as a spine only if a clearly identifiable enlarged head and narrow shaft were present; protrusions were scored as filopodia only if they were narrow, >3 µm in length, and failed to have an enlarged head. While in our initial analysis of protrusion length we measured all protrusions (Figure 2D), here we focused on protrusions that could be clearly defined as either dendritic spines or filopodia since EphBs have previously been shown to be important regulators of these structures. Consistent with the overall decrease in protrusion density using shRNA targeting EphB2, we found that EphB2 knockdown causes a reduction in both spine and filopodia density (Figure 3). Interestingly, while knockdown of EphB2 resulted in a 50% decrease in dendritic spine density in both apical and basal dendritic arbors (control, 0.083±0.010 spines/µm; apical EphB2 shRNA, 0.049±0.012 spines/µm; basal EphB2 shRNA, 0.048±0.012 spines/µm; p<0.05), knockdown only caused a significant decrease in filopodia density in basal dendrites (control, 0.050±0.010 filopodia/µm; apical EphB2 shRNA, 0.033±0.009 filopodia/µm; basal EphB2 shRNA, 0.020±0.007 filopodia/µm; p<0.05 for control vs knockdown in basal filopodia). Though there is a trend towards a reduction in filopodia density in apical dendrites as well, this change did not reach statistical significance (p = 0.23). Regardless, these findings raise the possibility that EphBs might have distinct roles in different regions of the dendritic arbor.

**Figure 4:**
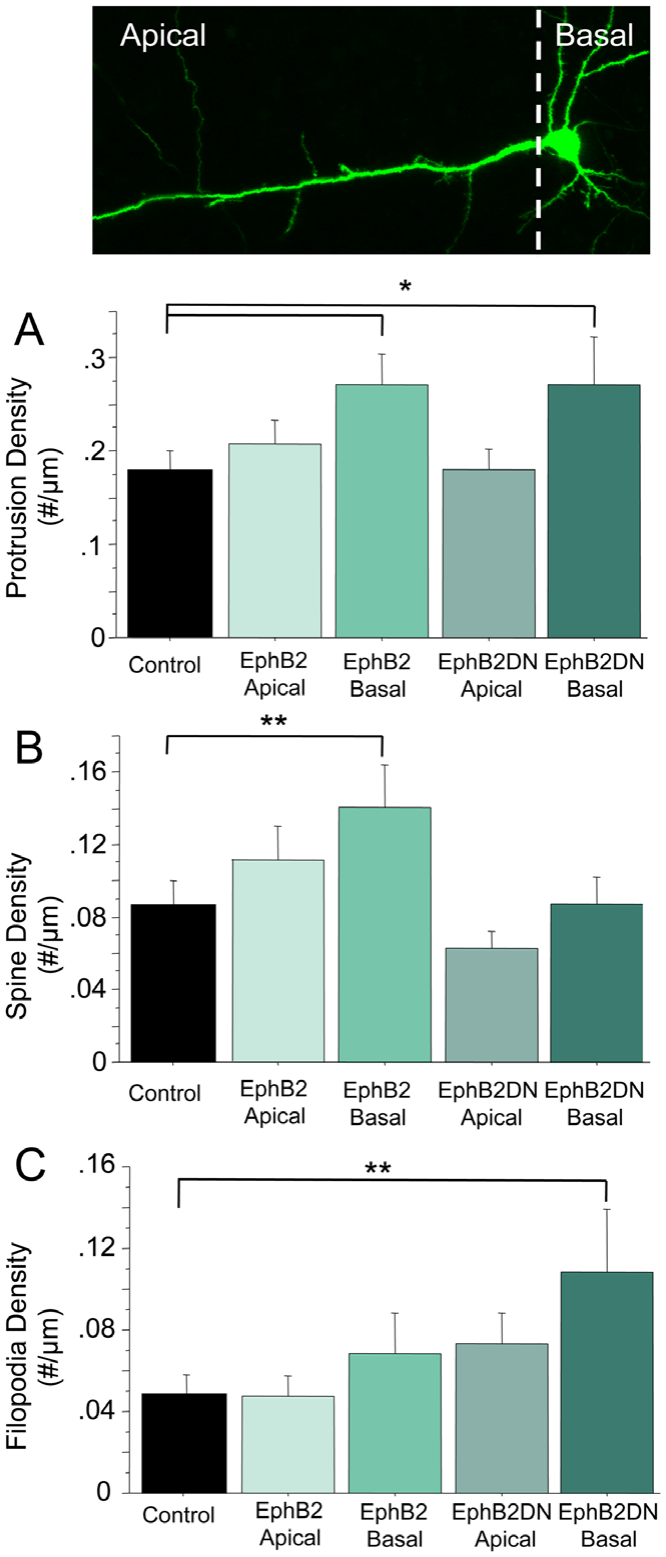
Preferential effect on protrusion structure in basal dendrites with overexpression of wild type or mutant EphB2. Top: Sample image of GFP-expressing cortical neuron in brain slice showing apical and basal portions of dendritic tree. A. Quantification of density of classifiable protrusions in the apical or basal portions of neurons transfected with the indicated construct. B. Quantification of spine density in the apical or basal portions of neurons transfected with the indicated construct. C. Quantification of filopodia density in the apical or basal portions of neurons transfected with the indicated construct. Control: n = 18 neurons; EphB2, apical and basal: n = 16 neurons; EphB2DN, apical and basal: n = 15 neurons; Error bars indicate standard error. Scale bar = 2 µm; *p<0.05. **p<0.03.

Although expression of EphB2 or EphB2DN did not result in an overall change in the number of protrusions, the expression of these constructs did cause changes in the average protrusion length in brain slice culture. To test whether expression of wild type EphB2 or EphB2DN preferentially impacts dendritic protrusions in apical or basal dendritic arbors, we examined the density of spines and filopodia in the dendrites of transfected cortical pyramidal neurons in brain slice culture. Overexpression of either EphB2 or EphB2DN resulted in an 50% increase of protrusion density specifically in basal dendrites when considering only those protrusions that are easily classified into a morphologic category (control, 0.181±0.019 protrusions/µm; apical EphB2, 0.208±0.027 protrusions/µm; basal EphB2, 0.271±0.034 protrusions/µm; apical EphB2DN, 0.181±0.022 protrusions/µm; basal EphB2DN, 0.271±0.051 protrusions/µm; p<0.05 for control vs. basal protrusion density) (Figure 4A). This significant change in protrusion density is revealed only by analyzing apical and basal dendrites independently. Consistent with the shortening of protrusion length with wild type EphB2 overexpression (Figure 2), transfection of EphB2 led to a ∼50% increase in density of dendritic spines compared to control, though only in basal dendrites (control, 0.087±0.013 spines/µm; apical EphB2, 0.112±0.019 spines/µm; basal EphB2, 0.141±0.024 spines/µm; apical EphB2DN, 0.063±0.010 spines/µm; basal EphB2DN, 0.087±0.015 spines/µm; p<0.03 for control vs. EphB2 overexpression basal spine density) (Figure 4B). In contrast, EphB2DN caused a selective doubling of filopodia density, once again only in the basal dendritic arbor (control, 0.049±0.009 filopodia/µm; apical EphB2, 0.048±0.010 filopodia/µm; basal EphB2, 0.068±0.020 filopodia/µm; apical EphB2DN, 0.073±0.016 filopodia/µm; basal EphB2DN, 0.109±0.030 filopodia/µm; p<0.03 for control vs. EphB2DN overexpression basal filopodia density) (Figure 4C). These results are not due to a more generalized effect on basal dendrite branching, as Sholl analysis demonstrated no significant changes in dendritic arbor complexity between control and experimental conditions (data not shown). To determine whether manipulation of any Eph receptor might result in changes in protrusion density, we also examined how overexpression of the EphA4 receptor impacts dendritic protrusions in cortical brain slice cultures. We chose EphA4 because it can be activated by binding either ephrin-As or ephrin-Bs [11], is found at synapses in mature brain [13], and regulates dendritic spine morphology in hippocampus [28]. In contrast to our findings with EphB2, overexpression of EphA4 had no effect on total protrusion density in apical or basal dendrites in cortical slices (total protrusions: control, 0.223±0.025 protrusions/µm; apical, 0.205±0.037 protrusions/µm; basal, 0.216±0.036 protrusions/µm). Moreover, we detected no changes in spine or filopodia density in either dendritic compartment (spines: control, 0.133±0.017 spines/µm; apical, 0.112±0.022 spines/µm; basal, 0.124±0.029 spines/µm; filopodia: control, 0.063±0.014 filopodia/µm; apical, 0.062±0.019 filopodia/µm; basal, 0.052±0.011 filopodia/µm; control n = 15, apical/basal n = 5). The inability of EphA4 to trigger changes in protrusion structure also indicates that binding of axonal ephrin-Bs alone is not sufficient to induce changes in filopodia and spines in cortical slices, as EphA4 binds ephrin-B family members in addition to ephrin-As [11]. Together, this data suggests a specific role for EphB2 signaling in dendritic protrusion structure of the basal tufts of cortical pyramidal neurons.

## Discussion

We examined how knockdown or overexpression in individual neurons of the EphB2 receptor tyrosine kinase affects the formation of dendritic spines and filopodia in cortical brain slice cultures with maintained cytoarchitecture. We find that knockdown of EphB2 reduces the number of dendritic protrusions, while overexpression of wild type or dominant negative EphB2 bidirectionally shift protrusion length without altering overall protrusion density. Interestingly, we also provide evidence that these functions are differentially regulated in the apical and basal dendritic arbors of cortical pyramidal neurons.

Overall, our findings are consistent with previous reports indicating that loss of EphB expression results in reduced numbers of dendritic protrusions and spines [16], [22]. By performing these experiments in organotypic brain slice culture, we show that modulation of EphB expression/signaling in individual neurons is sufficient to direct dendritic spine formation even in a more intact preparation. Analysis of dendritic protrusion length suggests that EphB signaling is an important element in the maturation of these structures, as overexpression of EphB2 results in a significant decrease in the length of these protrusions while a dominant negative EphB2 causes an increase in length. These results are likely to be of biological significance, as our data and other published results indicate that (1) EphB2 is expressed in cortex at this time in development [20], [27], (2) EphB2 is found at synapses [13], and (3) loss of EphBs from cortex results in abnormalities in synapse and spine formation [16], [22]. Overexpression of EphA4 does not cause similar changes in protrusion structure, further arguing against a nonspecific effect stemming from changes in Eph receptor expression levels.

We report that the predominant phenotype of blocking EphB signaling with the dominant negative construct (longer protrusions) is different than knockdown of EphB2 (fewer protrusions). Similarly, previous genetic experiments have demonstrated that EphB2-mediated forward signaling is required for normal spines in hippocampus [16]. Our observed differences between the effects of dominant negative EphB2 and EphB2 knockdown likely reflect the maintained extracellular interactions of EphB2DN, including binding of NMDARs and trans-cellular interactions with ephrin-Bs, without normal downstream signaling required to induce spines through signaling molecules such as PAK, Kalirin, or Tiam1 [14], [17], [26] or by inhibiting negative regulators of spine development such as Ephexin5 [29]. Importantly, trans-synaptic interactions with ephrin-Bs alone are likely not sufficient to drive changes in protrusion structure: overexpression of EphA4 – which binds some ephrin-Bs in addition to ephrin-As – does not appear to result in alterations in spine or filopodia density throughout the dendritic tree in cortical neurons at the ages examined. Another notable difference between our knockdown and overexpression data is the weaker preference for changes in basal dendrites with EphB2 knockdown in comparison to EphB2 or EphB2DN overexpression (Figures 3 and 4). Our EphB2 shRNA knockdown construct has been well characterized and found to be specific for EphB2, rescuable in multiple preparations, and to have no observable effects in neurons lacking EphBs [14]. Thus, it seems unlikely this result is nonspecific or due to off target effects, particularly as our results with EphB2 knockdown are consistent with published work in both dissociated neurons and in EphB triple knockout mice [14], [16], [22]. The data suggest that changes in EphB2 signaling and expression preferentially – but not exclusively – impact dendritic protrusions in the basal arbor.

Our finding that expression of either EphB2 or EphB2DN fails to induce changes in overall protrusion density, but instead appears to alter the proportion of protrusions that are spines or filopodia, indicates that forward signaling by EphB2 is critical for spine formation but not necessary for maintaining overall protrusion number in cortical slice cultures. This finding differs in some respects from previous work that demonstrated a change in protrusion density with expression of a dominant negative EphB2 construct [25]. These differences are likely a result of examining neurons in different regions of brain and in a more intact preparation. However, the core findings of a higher percentage of spines with increased EphB2 activation and a higher percentage of filopodia after disrupting EphB forward signaling is consistent across studies.

In sum, our data suggest that EphB2 may regulate contacts from a specific subpopulation of inputs or contacts that form on a particular portion of the dendritic arbor. Taking advantage of brain slices, which have preserved cortical layers and pyramidal cell polarity, we find that alterations in EphB2 function preferentially impact dendritic protrusions of basal dendrites in layer 2/3 and 5 neurons. Interestingly, basal dendrites of cortical pyramidal cells appear to receive strong excitatory glutamatergic inputs that are driven by NMDARs, as opposed to voltage-gated channels in much of the apical tree [9], [10]. Therefore, it is tempting to speculate how this surprising anatomical specificity may be linked to the ability of EphBs to interact with, cluster and regulate the function of the NMDAR [18], [21]. Regardless, these data begin to define a molecular mechanism that appears to regulate the formation of a subpopulation of dendritic spines. Future work will need to examine if loss of EphB2 results in functional changes corresponding to this apparent specificity, and whether other trans-synaptic proteins also play a role in dendritic spine formation in distinct subcellular patterns.

## Materials and Methods

All animal work was done in accordance with an animal protocol (#707892) that was approved by the University of Pennsylvania Institutional Animal Care and Use Committee (IACUC). Brain slices are made from P1–2 rat pups and placed into culture as described previously [22]. Briefly, 300 µm cortical brain slices (Vibratome, Warner Instruments) are made and placed in 0.4 µm Millicell Culture Inserts (Millipore) in 6-well plates. Slices are maintained in a humidified incubator with 5% CO_2_ at 37°C in 1 mL of stock solution medium containing 50 mL NB and supplements, 25 ml Hank's solution, 25 mL Horse Serum, 0.65 g dextrose, 1 mL 1 M HEPES, 1 mL P/S, at pH 7.2–7.3.

For immunostaining of brain sections, mice are transcardially perfused with ice-cold PBS followed by 4% paraformaldehyde in PBS. Brains are removed, post-fixed overnight in 4% paraformaldehyde at 4°C and incubated in 30% sucrose at 4°C until equilibrated. Brains are frozen for 1 min at −50°C in dry-ice cooled 2-methylbutane (Fisher Scientific) and stored at −80°C until sectioned. Either coronal or sagittal sections of 40 µm are cut using a freezing sliding microtome and processed as free-floating sections. Sections are blocked overnight at 4°C in PBS containing 5% horse serum and 0.4% Triton-X-100. Sections are incubated for 24 h in EphB2 anti-goat primary antibody (R&D Systems) diluted 1∶250 in the blocking buffer at 4°C with gentle agitation and then washed three times (10 min each) in 1×PBS. Secondary antibody (anti-goat Cy3, diluted 1∶250 in the blocking buffer) is applied for two hours at room temperature and sections are then washed 3× (10 minutes each) with PBS. Sections are mounted on slides and coverslipped using Hoechst containing Aqua-Mount (Fisher) medium to label nuclei. Images are collected on Leica wide field microscope.

Generation of the fEphB2, fEphB2_DN cDNA expression constructs and the EphB2 shRNAi are described previously [14], [18]. Transfection of cortical slices is performed after 1-2DIV using Helios Gene Gun (Bio-Rad). Briefly, 15–20 µg cDNA is coated to 6.25 mg of 1.6 µm gold particles in a solution with 0.01 mg/mL PVP, 0.05 M spermidine, 1 M CaCl_2_, and H_2_0, which is drawn into tubing and coated to the sides. Tubing is cut into cartridges, loaded into the Gene Gun, and gold particles are shot with high-pressure helium (120–140 psi) into cultured slices in inserts sitting on warmed agarose slabs. Successful delivery of gold particles can be visualized with dissection microscope, and transfection verified with fluorescence microscopy. Previous work has demonstrated a high rate (>90%) of co-transfection of constructs when multiple cDNA are coated onto the same gold particle [30].

For brain slice imaging, cultured slices are cut out from their insert at 4-7DIV, placed into ACSF at room temperature, and imaged with 40×/0.8 water immersion lens (Leica) on a two-photon laser confocal scanning microscope (Leica confocal microscope with Tsunami Ti:sapphire mode-locked laser tuned to 880–920 nm, pumped by a 10 W solid state laser source [Spectra Physics]). Images are acquired at 2× zoom in stacks with1.0 µm depth intervals. All images are acquired blind to experimental condition and analyzed blind to condition in NIH ImageJ. Significance between experimental conditions is determined by ANOVA. Control data are protrusion densities using both regions of the dendritic tree because there were no significant differences between any values in apical or basal regions of dendrites.

For morphology analysis in GFP-filled cells, individual protrusions were identified manually, blind to condition along at least 50 µm of dendrite. Protrusions with a well-developed mushroom-shaped head separated from the dendritic shaft by a distinct neck are scored as “spine.” Protrusions as long as a typical spine or longer with no definable head are scored as “filopodia-like.” All other protrusions, consisting mostly of thin and stubby protrusions, are scored as “other.”
